# Retraction: Chemopreventive Activity of *Ferulago angulate* against Breast Tumor in Rats and the Apoptotic Effect of Polycerasoidin in MCF7 Cells: A Bioassay-Guided Approach

**DOI:** 10.1371/journal.pone.0356047

**Published:** 2026-08-14

**Authors:** The PLOS One Editors

After publication of the Expression of Concern on this article [1,2], the authors have not provided the underlying data that support the published results. As such, PLOS cannot clarify the concerns about the results presented in Figs 6, 9, and 12 summarized in the Expression of Concern [2] and so the *PLOS One* Editors retract this article.

SZS did not agree with the retraction. HK responded but expressed neither agreement nor disagreement with the editorial decision. MF, SZM, MH, MR, MAA, HMA, and MIN either did not respond directly or could not be reached.
